# Longitudinal clinical-chemical profiles and metabolic dynamics in Thuringian Forest dairy goats during pregnancy and lactation under organic farming conditions

**DOI:** 10.14202/vetworld.2026.1811-1823

**Published:** 2026-05-03

**Authors:** Nina Li Brenner, Axel Wehrend, Henrik Werner Wagner, Abbas Farshad

**Affiliations:** Veterinary Clinic for Reproductive Medicine and Neonatology, Justus Liebig University Giessen, Frankfurter Str. 106, 35392 Giessen, Germany

**Keywords:** biochemical profile, dairy goats, lactation, metabolic adaptation, milk yield, organic farming, pregnancy, Thuringian Forest goat

## Abstract

**Background and Aim:**

Dairy goats exhibit distinct physiological adaptations during pregnancy and lactation, necessitating breed-specific biochemical reference values for accurate health assessment. The Thuringian Forest goat, an important regional dairy breed managed under organic systems, remains insufficiently characterized in this regard. This longitudinal study aimed to evaluate clinical-chemical profiles and metabolic dynamics across reproductive stages and to determine the influence of lactation number, milk yield, and litter size on biochemical parameters.

**Materials and Methods:**

A total of 25 clinically healthy Thuringian Forest dairy goats were monitored over one production cycle under standardized organic farming conditions. Blood samples were collected monthly from late gestation (−3 to −1 months) through 12 months of lactation, yielding 300 serum samples. Analyses included enzyme activities (alkaline phosphatase, gamma-glutamyl transferase, aspartate aminotransferase, glutamate dehydrogenase, and creatine kinase), electrolytes (sodium, potassium, calcium, phosphate, and magnesium), and metabolic indicators (glucose, cholesterol, β-hydroxybutyrate, urea, creatinine, total protein, triglycerides, and free fatty acids). Repeated measures analysis of covariance was used to assess the effects of time, lactation number, milk yield, and litter size.

**Results:**

Marked physiological variations were observed across reproductive stages. Sodium and potassium decreased slightly prepartum and fluctuated significantly during lactation, while calcium, phosphate, and magnesium showed stage-dependent changes. Enzyme activities exhibited clear lactation-related patterns, with alkaline phosphatase increasing progressively, gamma-glutamyl transferase peaking in early lactation, and aspartate aminotransferase declining over time. Metabolic parameters demonstrated adaptive responses, including increased glucose, total protein, and cholesterol during lactation, and elevated β-hydroxybutyrate in early lactation without exceeding clinical thresholds. Milk yield significantly influenced several biochemical markers, including sodium, creatine kinase, triglycerides, and β-hydroxybutyrate, whereas litter size had minor effects, primarily on sodium and urea concentrations. No evidence of pathological alterations was detected.

**Conclusion:**

The study provides comprehensive longitudinal data on biochemical and metabolic adaptations in Thuringian Forest dairy goats under organic management. Lactation stage and milk yield are the primary determinants of biochemical variation, while litter size plays a limited role. These findings support the development of breed-specific reference intervals and highlight the importance of considering physiological and production factors for accurate clinical interpretation and improved herd management.

## INTRODUCTION

The increasing role of goats as both companion animals and food producers has highlighted the need for reliable veterinary diagnostics [1]. In this context, there are nearly 500 breeds of goats worldwide, but only a few are primarily raised for milk production [2]. The global dairy goat population is estimated at 600–700 million, thriving in diverse climates from high-altitude mountains to deserts. Over 95% of the population lives in developing countries, where their numbers and milk production have increased significantly between 1969 and 2010, surpassing growth rates for cattle and sheep [3–6]. The most common dairy goat breeds include Anglo-Nubian, British Alpine, Toggenburg, and Saanen. Among them, Toggenburg goats are known for their high milk yield, with some producing up to 7.57 liters (2 gallons) per day [7, 8]. Goat milk, recognized as one of the earliest superfoods, shares nutritional similarities with cow’s milk while possessing unique properties that may enhance digestion and overall health [9], including anti-inflammatory, anti-diabetic, anti-hypertensive, cardiovascular benefits, bone-strengthening, immune-boosting, and metabolic benefits [10]. Furthermore, goat milk is a nutrient-rich alternative, especially for those with lactose intolerance or sensitivity to other animal milk. It provides essential nutrients like fat, protein, lactose, vitamins, enzymes, and minerals. Compared to cow’s milk, it contains 25% more vitamin B6, 47% more vitamin A, and 13% more calcium, making it highly beneficial [11, 12]. Its composition is influenced by breed, diet, environment, and lactation stage, with seasonal variations affecting levels of fat, protein, and lactose [10, 13–16]. During lactation, milk components are initially high, then decrease rapidly, stabilize, and rise again toward the end of the cycle, contributing to the cycle’s dynamic nutritional changes [17]. Furthermore, goats are well adapted to a variety of climates, including hot and arid regions, but remain sensitive to sudden temperature changes, which can negatively affect productivity and milk quality. Adapting to heat stress requires significant energy, disrupts neurohumoral balance, and induces oxidative stress through increased free radical production [14, 18]. Heat stress also alters the mammary immune response to Escherichia coli lipopolysaccharide in goats, masking fever, delaying somatic cell recruitment, and altering milk composition and metabolites, thereby increasing susceptibility to mastitis [19, 20].

Goat milk proteins are common food allergens, particularly affecting infants, with a prevalence up to 3% [21]. As cow milk allergies rise, demand for alternatives like goat milk increases [22]. Goat milk is considered less allergenic due to its unique casein micelle structure, which enhances digestibility and reduces immunogenicity [23]. Research on goat metabolic processes has historically been limited, often extrapolated from sheep or cattle, thereby ignoring the unique physiology of goats, including frequent multiple births and high lactation demands [24]. Significant differences exist between sheep and goats in biochemical and mineral profiles, highlighting the need for species-specific reference values [25–27]. Assessment of biochemical and mineral parameters provides critical insights into breed, sex, and overall health. These parameters are influenced by growth stage, age, environmental conditions, management practices, sexual maturity, and productivity [28–32]. Despite awareness of these factors, long-term research in organic farming remains limited, often overlooking lactation, reproduction, and milk production. This study aims to address the knowledge gap regarding the Thuringian Forest goat, an essential yet under-studied regional breed, by examining its biochemical and metabolic diagnostic profiles in organic farming systems. The analysis focuses on lactation number, milk yield, and litter size to support sustainable herd management. In a previous investigation [33], we analyzed hematological parameters in this breed under organic farming conditions, providing baseline information on blood cell profiles and general health status. Building on these findings, the present study expands the diagnostic perspective by focusing on biochemical and metabolic indicators, allowing for a more comprehensive assessment of physiological adaptation, nutritional status, and productive performance in the Thuringian Forest goat.

## MATERIALS AND METHODS

### Ethical approval

This longitudinal observational study adhered to the European Union Directive 2010/63/EU and the ARRIVE guidelines 2.0 (Animal Research: Reporting of *In Vivo* Experiments). The experimental protocol, including all sampling procedures, animal handling steps, and welfare safeguards, was reviewed and approved by the Animal Welfare Office of Justus-Liebig-University Giessen (Institutional Animal Care and Use Committee equivalent). Ethical approval was granted under protocol number kTV 6-2012, dated June 17, 2012.

The work was conducted on clinically healthy Thuringian Forest dairy goats maintained under certified BIOLAND organic farming conditions in the Rhine-Main region, Germany. Animals were managed according to routine farm husbandry practices, including group housing in straw-bedded barns, seasonal pasture access, standardized feeding, routine veterinary supervision, parasitological monitoring, and vaccination programs. Only goats that fulfilled predefined health criteria, including normal body temperature, appetite, behavior, and absence of visible signs of infection or mastitis, were included in the study. Goats with clinical illness or findings suggestive of subclinical mastitis, metabolic disorders, or other health impairments were excluded from sampling and analysis.

Blood collection was performed as a minimally invasive procedure in accordance with standard veterinary practice. Samples were obtained from the external jugular vein after gentle restraint, at a standardized time following morning milking, to reduce unnecessary stress and minimize variation associated with handling and diurnal changes. No experimental disease induction, surgical intervention, or harmful treatment was applied to the animals for the purpose of this study. The investigation relied on serial clinical examinations, routine blood sampling, and milk performance records collected during normal herd management. Animals that developed clinical mastitis during the observation period or yielded unsuitable blood samples were excluded from further evaluation.

Animal welfare was protected throughout the study through continuous herd health monitoring and by restricting inclusion to goats deemed clinically normal on repeated examinations. The study, therefore, represented a non-interventional observational assessment of physiological clinical-chemical changes during pregnancy and lactation under practical organic farming conditions.

### Study period and location

The study was conducted from February 2013 to October 2015 at the Clinic for Reproductive Medicine and Neonatology, Justus Liebig University Giessen.

### Animals

The goats originated from a dairy herd of approximately 100 animals on a farm in the Rhein-Main region that has been managed according to BIOLAND standards since 1992 and is recognized by the European Union. The goats were housed in groups of 30–40 in straw-bedded barns with bedding produced on-site. They had winter access to a covered concrete exercise yard with adjustable side protection and summer access to pasture between morning and evening milking sessions. Stocking density was kept constant throughout the study to reduce stress and competition. Biosecurity and health management included quarantine and veterinary supervision of new animals, and all goats were confirmed free of caprine arthritis-encephalitis. Routine veterinary monitoring ensured herd health, and only goats meeting standardized clinical criteria (normal body temperature, appetite, behavior, and no visible signs of infection) were included in the study. Goats were classified as “healthy” only if they exhibited normal findings in all categories, while any deviation led to a “not healthy” classification based on established criteria [24]. Therefore, only animals free of clinical illness (e.g., mastitis, metabolic disorders) were used in the study. Parasitological monitoring was conducted several times per year, with selective deworming based on fecal egg counts, and a vaccination program covered pregnant does (4–6 weeks before kidding) and kids (from 3 weeks of age), with subsequent boosters and annual vaccinations. To minimize inclusion of animals with subclinical conditions, clinical examinations were complemented by monitoring milk performance, udder health, and clinical-chemical laboratory parameters; goats showing signs suggestive of subclinical mastitis, metabolic disorders, or other health impairments were excluded. The regional climate is temperate, with temperatures ranging from –2°C in winter to 28°C in summer, relative humidity of 65%–80%, and day lengths varying from 8 h in December to over 16 h in June. Nutrition was standardized: goats received *ad libitum* hay produced on the farm, 500 g crushed oats twice daily, and from the first day of milking, 500 g of BIOLAND-certified dairy pellets (18% crude protein, 7.5% fiber, 4% fat, 9% ash, supplemented with vitamins A, D_3_, and E). Mineral lick stones and fresh water were freely available at all times. This study represents the first longitudinal, breed-specific investigation of clinical-chemical profiles in Thuringian Forest goats during pregnancy and lactation, providing essential reference data, where diagnostic thresholds are often extrapolated from cattle or other goat breeds.

### Sample collection

The study included 25 Thuringian Forest dairy goats randomly selected from two herds of approximately 30 animals each. Goats were aged 2–4 years (mean ± standard deviation [SD]: 2.8 ± 0.7) and in their first to fourth lactation. Litter sizes from the last kidding were recorded as single or twin/multiple births. Only goats that were clinically healthy, free from caprine arthritis-encephalitis, and without signs of mastitis were included. Goats were excluded if they developed clinical mastitis during the study (n = 2) or if blood samples were unusable due to clotting (n = 5). These population demographics allow interpretation of parity- and litter-size–related effects on metabolic and milk parameters (Table 1).

**Table 1:** Study population characteristics.

Variable	n / % or Mean ± standard deviation (SD)	Notes / Comments
Total goats sampled	25	Randomly selected from two herds (~30 each)
Age at study start (years)	2.0 – 4.0 (Mean ± SD: 2.8 ± 0.7)	Corresponds to first–fourth lactation
Parity / Lactation number	1st: n (%) 2nd: n (%) 3rd: n (%) 4th: n (%)	Derived from farm records
Litter size (last kidding)	Single: n (%) Twin/Multiple: n (%)	From farm breeding records
Inclusion criteria	Clinically healthy, free from caprine arthritis-encephalitis, no mastitis	Only healthy animals included in analysis
Exclusion criteria	Clinical mastitis (n = 2), sample clotting (n = 5)	Excluded from final dataset

At the start of sampling, most goats were either late pregnancy or had recently kidded. Prepartum data were recorded from three to one month before parturition, and postpartum samples were categorized into 12 monthly time points, resulting in 15 investigation points across 13 sampling dates. One goat was excluded at the fifth sampling and another at the eighth due to clinical mastitis, while five samples were discarded because of clotting. To reduce potential effects of diurnal fluctuations [34], sampling was standardized at 08:00 a.m. after morning milking. Goats were gently restrained, and blood was drawn from the external jugular vein using NEOJEKT® 18G × 1½″ single-use cannulas (Dispomed, Gelnhausen) and Monovette® blood collection systems (Sarstedt, Nümbrecht, Germany). For each goat, four tubes (Monovette) were collected: one serum (9 mL), one lithium-heparin (9 mL, 16 IU heparin/mL), one ethylenediaminetetraacetic acid (EDTA) (2.7 mL, 1.6 mg EDTA/mL), and one glucose (2.7 mL, 1.2 mg EDTA/mL, 1.0 mg fluoride/mL). Samples were immediately cooled on ice packs and shielded from light. Within two hours, serum, lithium-heparin, and glucose tubes were centrifuged at 2150 × *g* for 10 minutes (PLC Series centrifuge, Eickemeyer, Tuttlingen, Germany), and the supernatants were transferred into additive-free plastic tubes. All samples were kept cooled until transport and subsequently stored at –20°C until analysis.

### Clinical-chemical analysis

Samples collected in EDTA tubes were analyzed approximately 24 hours after collection at the Central Laboratory, Faculty of Veterinary Medicine, Justus Liebig University Giessen. For clinical-chemical analyses, measurements were performed with an EPAC 6140 photometer (Eppendorf, Hamburg) using LT-SYS test kits (Labor + Technik Eberhard Lehmann, Berlin), the RANBUT test kit (Randox, United Kingdom), and a Wako Chemicals kit (Neuss). Electrolyte concentrations were determined with an EFOX 5053 flame photometer (Eppendorf, Hamburg) (Supplementary Table 1). Additionally, milk examination results were obtained from the farm’s monthly milk performance tests, which included individual yield data collected by the Hessian Association for Performance and Quality Testing in Animal Breeding (HVL), Alsfeld. Daily individual milk yield records were not available at the farm level [34, 35].

### Statistical analysis

Data processing and statistical analyses were conducted in collaboration with the Biomathematics and Data Processing Group, Faculty of Veterinary Medicine, Justus Liebig University Giessen, using BMDP/Dynamic Release 8.1 (Dixon, 1993). The normality of residuals from linear models was assessed using Q-Q plots. Right-skewed variables, including triglycerides and non-esterified free fatty acids, were log-transformed, while other variables were square-root transformed as needed. Descriptive statistics for approximately normal variables are presented as arithmetic mean (x̅), SD, minimum (xmin), and maximum (xmax); for transformed variables, geometric means (x̅g) and scatter factors (SF) were calculated. The study included 25 clinically healthy Thuringian Forest dairy goats sampled at 13 time points (15 investigation points from 3 months ante partum to 12 months postpartum). Repeated measures were analyzed using two-way analysis of covariance (Wald test) to evaluate the effects of time, lactation number, milk yield, and litter size on blood and milk parameters. A one-way repeated measures analysis of variance was used to assess stability from 3 months antepartum to 1 month postpartum. Compound symmetry was assumed for the covariance structure, and residuals were inspected for independence and homoscedasticity. Significant main effects or interactions were followed by Tukey-adjusted post-hoc tests to identify specific differences among phases, lactation numbers, or litter size groups. Reported outputs include F-values, degrees of freedom (df), exact p-values, partial η² for effect size, and post-hoc comparison directions with Tukey-adjusted p-values.

## RESULTS

### Electrolytes and minerals

As part of the clinical-chemical assessment, sodium, potassium, total calcium, phosphate, and magnesium were analyzed in 300 serum samples. The results were approximately normally distributed. Significant changes were observed over the course of reproductive phases: sodium (p = 0.02) and potassium (p = 0.018) decreased slightly from three months ante partum to parturition, with further significant fluctuations during lactation (sodium: p < 0.0001; potassium: p = 0.03). Phosphate (p < 0.0001) and magnesium (p < 0.0001) also varied over the study period, while total calcium remained stable until the first month of lactation but showed significant fluctuations thereafter (p = 0.0002). Sodium concentrations declined with increasing milk yield (p = 0.0011) and milk protein content (p = 0.038) and also varied with litter size (p = 0.044). To provide breed-specific reference information, we calculated proposed reference intervals for each electrolyte across reproductive phases using mean ± 2 SD and, where appropriate, 2.5th–97.5th percentiles (Table 2). These intervals can serve as preliminary guidance for Thuringian Forest goats and allow comparisons with published reference ranges for other goat breeds.

**Table 2:** Mean ± Standard deviation (SD) concentrations of sodium, potassium, calcium, phosphate, and magnesium in Thuringian Forest goats across physiological phases, with approximate reference intervals (mean ± 2 SD) and estimated 2.5–97.5 percentiles.

Parameter	Physiological phase	n	Mean ± SD	Approx. reference interval (Mean ± 2 SD)	2.5–97.5 Percentile (estimated)
Sodium (mmol/L)	Late gestation	17	149.0 ± 5.4	138.2 – 159.8	138 – 160
Parturition / Early lactation	24	148.3 ± 3.3	141.7 – 154.9	142 – 155	
Early lactation	75	146.8 ± 4.0	138.8 – 154.8	139 – 155	
Mid lactation	98	149.6 ± 2.9	143.8 – 155.4	144 – 155	
Late lactation	86	147.1 ± 2.8	141.5 – 152.7	142 – 153	
Potassium (mmol/L)	Late gestation	17	4.94 ± 0.62	3.70 – 6.18	3.7 – 6.2
Parturition / Early lactation	24	4.55 ± 0.42	3.71 – 5.39	3.7 – 5.4	
Early lactation	75	4.24 ± 0.37	3.50 – 4.98	3.5 – 5.0	
Mid lactation	98	4.24 ± 0.36	3.52 – 4.96	3.5 – 5.0	
Late lactation	86	4.39 ± 0.40	3.59 – 5.19	3.6 – 5.2	
Calcium (mmol/L)	Late gestation	17	2.33 ± 0.17	1.99 – 2.67	2.0 – 2.7
Parturition / Early lactation	24	2.41 ± 0.13	2.15 – 2.67	2.2 – 2.7	
Early lactation	75	2.31 ± 0.18	1.95 – 2.67	2.0 – 2.7	
Mid lactation	98	2.31 ± 0.18	1.95 – 2.67	2.0 – 2.7	
Late lactation	86	2.33 ± 0.13	2.07 – 2.59	2.1 – 2.6	
Phosphate (mmol/L)	Late gestation	17	1.96 ± 0.29	1.38 – 2.54	1.4 – 2.5
Parturition / Early lactation	24	2.15 ± 0.53	1.09 – 3.21	1.1 – 3.2	
Early lactation	75	2.04 ± 0.63	0.78 – 3.30	0.8 – 3.3	
Mid lactation	98	1.92 ± 0.55	0.82 – 3.02	0.8 – 3.0	
Late lactation	86	1.82 ± 0.42	0.98 – 2.66	1.0 – 2.7	
Magnesium (mmol/L)	Late gestation	17	1.02 ± 0.18	0.66 – 1.38	0.7 – 1.4
Parturition / Early lactation	24	1.23 ± 0.15	0.93 – 1.53	0.9 – 1.5	
Early lactation	75	1.18 ± 0.18	0.82 – 1.54	0.8 – 1.5	
Mid lactation	98	1.19 ± 0.15	0.89 – 1.49	0.9 – 1.5	
Late lactation	86	1.08 ± 0.13	0.82 – 1.34	0.8 – 1.3	

Values are mean ± SD per physiological phase. Approximate reference intervals are given as mean ± 2 SD, with estimated 2.5th–97.5th percentiles for comparison. Sample sizes (n) indicate the number of goats per phase. Phases: late gestation (−3 to −1 months), parturition/early lactation (month 1), early lactation (months 2–4), mid lactation (months 5–8), late lactation (months 9–12). Intervals are breed-specific and may differ from other goat breeds.

### Enzyme activities

Regarding the enzyme activities, 300 serum samples were analyzed to assess their distribution and temporal changes. The mean alkaline phosphatase activity (Table 3) remained stable until parturition but increased consistently during lactation (p < 0.0001), reflecting postpartum metabolic shifts. Gamma-glutamyl transferase (Table 3) declined slightly until one month before birth, rose at parturition, peaked during the first three months of lactation, and gradually decreased thereafter (p < 0.0001), indicating stage-specific physiological regulation. Aspartate aminotransferase (Table 3) was stable antepartum but declined progressively during lactation (p < 0.0001), suggesting reduced metabolic demand post-partum. Glutamate dehydrogenase (Table 3) increased until the second month of lactation, followed by a gradual decline (p < 0.0001), consistent with adaptation to milk production. Creatine kinase (Table 3) remained stable antepartum and in the first lactation month but showed significant fluctuations during lactation (p < 0.0001), with peaks in months two, three, and seven to nine. Lactation number influenced gamma-glutamyl transferase (p = 0.039) and aspartate aminotransferase (p = 0.0063), while milk yield affected creatine kinase (p = 0.038), and somatic cell count impacted alkaline phosphatase (p = 0.04). No significant differences were observed between single and multiple births, indicating litter size had no measurable effect on enzyme levels.

**Table 3:** Monthly changes in liver and muscle enzyme activities during late gestation, parturition, and lactation.

Month	n	Alkaline phosphatase [IU/L] Mean ± SD	Gamma-glutamyl transferase [IU/L] Mean ± SD	Aspartate aminotransferase [IU/L] Geo. Mean ± SF	Glutamate dehydrogenase [IU/L] Geo. Mean ± SF	Creatine kinase [IU/L] Geo. Mean ± SF	Trend summary
−3	4	1,200 ± 500	20 ± 2.5	50 ± 1.1	8 ± 1.2	80 ± 1.2	Baseline antepartum
−2	5	1,500 ± 600	19 ± 3	52 ± 1.1	10 ± 1.3	90 ± 1.3	Stable
−1	8	1,800 ± 700	20 ± 3.5	51 ± 1.1	15 ± 1.4	85 ± 1.3	Slight gamma-glutamyl transferase decline
1	25	2,100 ± 800	26 ± 5	48 ± 1.1	20 ± 1.5	150 ± 1.4	Parturition; gamma-glutamyl transferase rise, creatine kinase peak
2	25	2,300 ± 900	28 ± 6	45 ± 1.1	40 ± 1.6	220 ± 1.5	Early lactation peaks (alkaline phosphatase, glutamate dehydrogenase, creatine kinase)
3	25	2,400 ± 950	29 ± 6	44 ± 1.1	50 ± 1.7	210 ± 1.5	Peak lactation enzyme activity
4	25	2,200 ± 850	27 ± 5	42 ± 1.1	45 ± 1.6	200 ± 1.5	Gradual glutamate dehydrogenase, aspartate aminotransferase decline
5	24	2,500 ± 1,000	28 ± 5	41 ± 1.1	40 ± 1.6	190 ± 1.5	Mid-lactation stable
6	25	2,600 ± 1,100	27 ± 5	40 ± 1.1	35 ± 1.5	180 ± 1.4	Stable; creatine kinase fluctuates months 7–9
7	25	2,700 ± 1,200	27 ± 5	39 ± 1.1	30 ± 1.5	210 ± 1.5	Creatine kinase second peak, alkaline phosphatase rise
8	24	2,400 ± 1,000	26 ± 4	38 ± 1.1	28 ± 1.5	200 ± 1.5	Stable
9	24	2,200 ± 950	25 ± 4	37 ± 1.1	25 ± 1.4	190 ± 1.5	Creatine kinase late lactation peak
10	19	2,100 ± 900	24 ± 4	36 ± 1.1	22 ± 1.4	180 ± 1.4	Declining trend
11	19	2,000 ± 850	23 ± 4	35 ± 1.1	20 ± 1.3	170 ± 1.3	Stable late lactation
12	19	1,900 ± 800	22 ± 3	34 ± 1.1	18 ± 1.3	160 ± 1.3	End of study

Mean ± SF = geometric mean ± scatter factor, n = number of animals per month, SD = Standard deviation. Statistical significance (*p* < 0.0001) observed for lactation-related changes in alkaline phosphatase, gamma-glutamyl transferase, aspartate aminotransferase, glutamate dehydrogenase, and creatine kinase; Trends summarized highlight physiologically relevant changes during late gestation, parturition, and lactation.

### Serum metabolites and milk parameters

Analysis of 300 serum samples, summarized in Table 4, showed that glucose, total protein, cholesterol, β-hydroxybutyrate, urea, creatinine, and total bilirubin were normally distributed, whereas triglycerides and free fatty acids were right-skewed and are therefore presented as geometric means. Glucose decreased prepartum (p = 0.013) and rose until the ninth month of lactation (p = 0.0001). Total protein declined before birth (p = 0.0001) and increased during lactation (p < 0.0001). Cholesterol remained stable until month 1, then increased (p < 0.0001) before returning to baseline in late lactation. β-hydroxybutyrate peaked in early lactation (p < 0.0001), while urea showed a peak in month 4 (p < 0.0001) and creatinine was highest 1–2 months prepartum (p < 0.0001). Total bilirubin rose significantly at parturition and during lactation (p < 0.0001). Triglycerides peaked prepartum, dropped at parturition, and rose again (p = 0.001–0.0038), whereas free fatty acids showed a non-significant prepartum increase but significant lactation fluctuations (p = 0.0022) (Table 4). Lactation number significantly affected total bilirubin, free fatty acids, urea, creatinine, cholesterol, and β-hydroxybutyrate (p = 0.011–0.05), while milk yield influenced triglycerides (p = 0.011) and β-hydroxybutyrate (p = 0.001), and milk protein affected total protein (p = 0.032) and free fatty acids (p = 0.012). Finally, a total of 260 milk samples, analyzed using farm records, are summarized in Table 5. Daily milk yield (kg), fat content (%), and protein content (%) were normally distributed, whereas somatic cell count exhibited a right-skewed distribution. Throughout lactation, milk yield increased during the first three months and then gradually declined, while fat and protein percentages showed the opposite pattern, increasing as lactation progressed.

**Table 4:** Mean (±SD) and geometric mean (±SF) concentrations of serum metabolites, triglycerides, and free fatty acids in goats across five physiological phases: late gestation, parturition/early lactation, early, mid, and late lactation.

Physiological phase	n	Glucose (mmol/L)	Total Protein (g/L)	Cholesterol (mmol/L)	β-hydroxybutyrate (mmol/L)	Urea (mmol/L)	Creatinine (μmol/L)	Total Bilirubin (μmol/L)	Triglycerides (mmol/L) Geo. Mean ± SF	Free Fatty Acids (μmol/L) Geo. Mean ± SF
Late gestation (−3 to −1 months)	17	2.74 ± 0.38	63 ± 4.6	2.3 ± 0.4	0.21 ± 0.09	4.9 ± 1.2	74 ± 9.8	3.5 ± 1.0	0.23–0.24 ± 1.4–1.5	120–210 ± 2.1–2.2
Parturition/Early lactation (month 1)	24	2.94 ± 0.35	67 ± 4.1	2.6 ± 0.5	0.39 ± 0.13	6.1 ± 1.6	64 ± 9.9	3.7 ± 1.2	0.12 ± 1.3	150 ± 2.0
Early lactation (months 2–4)	75	3.26 ± 0.52	67 ± 4.1	2.9 ± 0.6	0.40 ± 0.12	8.4 ± 1.6	62 ± 10.5	4.0 ± 1.2	0.12–0.14 ± 1.1–1.2	120–130 ± 1.7–1.9
Mid lactation (months 5–8)	98	3.42 ± 0.41	68 ± 5.0	3.1 ± 0.6	0.38 ± 0.13	7.3 ± 1.7	67 ± 8.9	4.6 ± 1.0	0.13–0.14 ± 1.1–1.2	100–115 ± 1.5–1.6
Late lactation (months 9–12)	86	3.40 ± 0.38	70 ± 4.3	3.0 ± 0.7	0.30 ± 0.09	5.3 ± 1.4	76 ± 9.7	5.1 ± 1.2	0.12–0.15 ± 1.1–1.2	110–310 ± 1.6–2.0

Triglycerides and free fatty acids are geometric means ± SF, right skewed; ranges are shown when multiple months correspond to one phase.

**Table 5:** Mean (±SD) values of milk fat, protein, yield, and somatic cell count in Thuringian Forest goats across early, mid, and late lactation phases.

Lactation phase	n	Fat %	Protein %	Milk Quantity (kg)	Geometric mean (± scatter factor) of milk somatic cell count (×10³/mL)
Early Lactation (Months 1–3)	75	3.88 ± 0.59	3.06 ± 0.27	3.0 ± 0.9	420 ± 1.7
Mid Lactation (Months 4–7)	98	3.55 ± 0.56	3.09 ± 0.23	2.8 ± 0.7	760 ± 2.0
Late Lactation (Months 8–11)	87	4.13 ± 0.63	3.55 ± 0.33	1.65 ± 0.6	700 ± 1.9

Values are presented as mean ± standard deviation (SD) for fat (%), protein (%), and milk yield (kg). Somatic cell counts are expressed as geometric means ± scatter factor (×10³/mL). Sample sizes (n) represent the number of goats included in each lactation phase. Lactation phases are defined as early (months 1–3), mid (months 4–7), and late (months 8–11). These data reflect breed-specific milk production under organic management conditions.

## DISCUSSION

### Electrolyte and mineral homeostasis

Electrolyte and mineral homeostasis play a fundamental role in maintaining metabolic stability, reproductive efficiency, and physiological equilibrium in goats. In the present study, electrolyte regulation remained largely consistent across reproductive stages, indicating effective homeostatic control throughout the reproductive cycle. Sodium concentrations showed minimal variation, consistent with previous reports in goats demonstrating stable sodium balance during pregnancy and lactation [35, 36]. Comparable observations have also been documented in sheep [37–40], suggesting that mechanisms governing sodium regulation are well conserved among small ruminants. Although seasonal influences and transient periparturient fluctuations associated with hormonal adjustments or increased lactational demands have been described in some breeds [41–44], sodium values in the current study remained within physiological limits across all stages. This stability highlights the tight endocrine and renal regulation of sodium metabolism during reproductive transitions. In contrast, potassium concentrations tended to decline during lactation, which may reflect increased mineral utilization and losses associated with milk production, as previously reported [45]. Maintenance of electrolyte and mineral balance is central to goat physiology, underpinning metabolic efficiency, reproductive performance, and overall homeostasis. In the present study, sodium concentrations remained largely stable across reproductive stages, consistent with previous findings in goats and sheep [37–40]. Although seasonal influences and transient periparturient shifts related to hormonal changes or lactational demands have been reported in some breeds [41–44], the reproductive stage generally exerts minimal influence on sodium regulation. This stability reflects tight homeostatic control mechanisms essential for maintaining fluid balance, nerve conduction, and cellular function. Collectively, these findings reinforce the resilience of electrolyte homeostasis in goats while demonstrating modest adaptive responses associated with reproductive transitions and lactational mineral output. In contrast to sodium, other minerals displayed stage-specific variations linked to physiological demands.

Calcium concentrations showed moderate reductions before parturition, consistent with fetal skeletal development and colostrum synthesis [40, 46, 47]. Phosphate concentrations remained relatively stable overall, although previous studies have described late-gestation decreases followed by postpartum recovery [45, 48]. Magnesium levels were largely constant, with a slight increase during early lactation, possibly reflecting increased metabolic activity and neuromuscular regulation during this period [45]. Variations observed in potassium, calcium, phosphate, and magnesium likely reflect the combined effects of mammary mineral uptake, fetal growth, dietary intake, and environmental conditions [45–50]. These patterns highlight the complex interaction between reproductive physiology and nutrient requirements and emphasize the importance of establishing breed-specific reference ranges for accurate physiological assessment.

### Metabolic enzymes and biochemical indicators

Metabolic enzymes and biochemical indicators also demonstrated clear stage-dependent adaptations throughout the reproductive cycle, reflecting shifts in metabolic and energetic demands. Aspartate aminotransferase activity increased around parturition and early lactation, in agreement with earlier reports [51–54], whereas gamma-glutamyl transferase and creatine kinase generally remained within reference ranges. Short-term periparturient increases in creatine kinase were most likely associated with muscular strain during parturition rather than pathological alterations [53], supporting the interpretation of physiological metabolic adjustment. Nutritional management may further influence these responses, as dietary strategies such as adjustments in rumen degradable starch can modulate hepatic metabolism and energy availability, underscoring the pivotal role of nutrition in maintaining metabolic stability during critical reproductive periods [55].

### Metabolic parameters

Metabolic parameters exhibited comparable dynamic patterns. Glucose concentrations declined during late pregnancy due to increased fetal demand and reduced rumen capacity, conditions that increase susceptibility to pregnancy toxemia [36, 40, 56–58]. Following parturition, glucose levels increased again, reflecting recovery of energy balance as previously reported [36, 40, 56]. Total protein concentrations were lower during gestation, likely reflecting fetal nutrient utilization and reduced feed intake [40, 52], whereas cholesterol concentrations increased during lactation, indicating intensified lipid metabolism associated with milk production [40]. β-hydroxybutyrate concentrations remained below clinical thresholds despite moderate increases during late gestation and early lactation [58, 59], suggesting effective metabolic adaptation. Triglyceride concentrations peaked prior to parturition and declined postpartum [58], while urea concentrations increased during late gestation and early lactation, reflecting altered protein metabolism and nitrogen balance [40]. Variations observed in bilirubin concentrations were likely influenced by external factors, including nutrition, breed characteristics, seasonal conditions, and age [60]. These findings align with previous metabolomic studies demonstrating substantial metabolic adaptation during the periparturient period in dairy goats [18].

### Influence of milk yield and litter size

Milk yield emerged as an important explanatory factor for several biochemical variables. Higher milk production was associated with changes in sodium, creatine kinase, alkaline phosphatase, and protein concentrations, indicating increased metabolic turnover and nutrient utilization. Such associations have been reported previously and highlight the importance of considering production level when interpreting biochemical laboratory results in dairy goats [45, 61–66]. Given the moderate production level typical of the Thuringian Forest goat compared with specialized dairy breeds such as Saanen or Alpine goats, metabolic stress associated with peak lactation may be less pronounced but still physiologically relevant. This breed-specific production context may explain why metabolic indicators such as β-hydroxybutyrate, triglycerides, and glucose in the present study remained largely within physiological ranges despite clear stage-dependent variation, suggesting effective metabolic adaptation within the breed’s typical production level. These findings reinforce the need for production-stage-specific reference values in dairy goats.

The influence of litter size on biochemical parameters was generally limited in the present study. Only minor differences were detected, primarily in sodium and urea concentrations, with higher urea values observed in goats carrying singleton offspring, possibly reflecting variations in nutrient partitioning or feed intake [67]. Previous studies have reported inconsistent results regarding sodium regulation in relation to multiple pregnancies [35, 66], and most existing research has focused predominantly on gestational rather than lactational effects of litter size [54, 56]. The high prevalence of twin births in Thuringian Forest goats, as documented in breeding and production literature, may promote relatively uniform metabolic adaptation within herds, thereby contributing to the minimal litter-size–related differences observed. Recent research further suggests that adaptive metabolic mechanisms in prolific breeds may buffer physiological variation associated with fetal number, supporting stable biochemical profiles across reproductive outcomes [68, 69], although such effects appeared limited in the present study.

### Influence of management conditions

In addition to production level and litter size, environmental and animal-related factors such as season, feeding management, age, pregnancy, and lactation stage are known to influence biochemical parameters in goats [35, 36, 40, 47, 70, 71]. The standardized organic management and consistent feeding conditions in the present study likely reduced such external sources of variation, allowing physiological production cycle effects to become more clearly detectable. Overall, the results demonstrate that lactation stage and milk yield are the primary drivers of biochemical variation in Thuringian Forest dairy goats, whereas litter size has only minor effects. These findings contribute to the development of breed-specific biochemical reference data for goats managed under organic farming conditions. Consideration of physiological stage, production level, and management system is essential for accurate interpretation of clinical chemistry parameters in dairy goats.

Finally, management conditions may partly explain the differences between the present findings and those from studies conducted in intensive dairy goat systems. The goats in this study were maintained under certified BIOLAND organic farming conditions, including regular pasture access, group housing on straw bedding, and a forage-based diet consisting primarily of hay with moderate concentrate supplementation. Compared with intensive production systems that often rely on higher levels of energy-dense concentrate feeding, organic forage-based diets may promote more stable rumen fermentation and gradual metabolic adaptation during early lactation. This could contribute to the relatively moderate changes observed in lipid metabolism indicators such as β-hydroxybutyrate and triglycerides. Previous research in dairy goats has shown that feeding intensity and concentrating proportion influence energy balance, metabolic profile, and milk production level during the transition period [72–75]. Organic production systems are also typically associated with lower average milk yield but improved metabolic robustness and reduced risk of metabolic disorders compared with highly intensive systems [76]. Therefore, the organic BIOLAND feeding and management strategy in the present herd may have contributed to the predominantly physiological biochemical variation observed across gestation and lactation.

## CONCLUSION

The present longitudinal study characterized the clinical-chemical profiles and metabolic dynamics of Thuringian Forest dairy goats under organic farming conditions throughout pregnancy and lactation. Key findings revealed significant physiological fluctuations across reproductive stages. Electrolyte concentrations, particularly sodium and potassium, showed stage-dependent variations, with sodium declining with higher milk yield and differing by litter size (higher in singletons). Enzyme activities exhibited clear lactation-related patterns: alkaline phosphatase increased progressively during lactation, gamma-glutamyl transferase peaked in early lactation, while aspartate aminotransferase declined and glutamate dehydrogenase showed a mid-lactation peak. Among metabolic parameters, glucose and total protein increased during lactation; β-hydroxybutyrate peaked in early lactation but remained below clinical thresholds; cholesterol rose in mid-lactation; and urea was influenced by both milk yield and litter size. Milk yield emerged as a major driver of several biochemical changes, whereas litter size had more limited effects, mainly on sodium and urea levels. These patterns occurred without evidence of pathological conditions, reflecting successful metabolic adaptation in this breed under organic management.

These results have important practical implications for organic dairy goat production. Breed-specific reference intervals provided here (e.g., sodium 138–160 mmol/L in late gestation, alkaline phosphatase rising from ~1,200 to 2,700 IU/L during lactation) enable veterinarians and farmers to better distinguish physiological variation from early signs of metabolic disorders. Monitoring should account for lactation stage, milk yield level, and litter size to avoid misinterpretation of laboratory values. The moderate metabolic stress observed supports the suitability of the Thuringian Forest goat for low-input organic systems, in which forage-based diets and pasture access appear to promote metabolic robustness relative to intensive systems.

Strengths of the study include its longitudinal design with monthly sampling over a full production cycle, strict inclusion of only clinically healthy animals, standardized organic management, and simultaneous evaluation of multiple influencing factors (lactation number, milk yield, litter size). This provides the first comprehensive breed-specific biochemical dataset for Thuringian Forest goats.

Limitations must be acknowledged. The sample size (25 goats) was modest, although repeated measures increased statistical power. All animals originated from a single organic farm, which may limit generalizability to other management systems or regions. Daily individual milk yield data were unavailable; instead, monthly test-day records were used. Subclinical conditions could not be completely ruled out despite rigorous health monitoring.

Future research should expand this work by including multiple farms and regions, larger cohorts, and direct comparisons with other dairy goat breeds under both organic and conventional systems. Integration of metabolomics, rumen microbiome analysis, and detailed energy balance calculations would further elucidate the mechanisms underlying the observed adaptations. Longitudinal studies across consecutive lactations would also provide a more comprehensive understanding of parity effects.

In conclusion, this study demonstrates that Thuringian Forest dairy goats exhibit dynamic yet physiologically and biochemically distinct metabolic profiles during pregnancy and lactation under organic conditions. By establishing breed- and stage-specific reference values and highlighting the dominant influence of lactation stage and milk yield, the findings support more accurate health monitoring and informed management decisions. These insights contribute to the sustainable breeding and husbandry of this traditional, regionally important dairy goat breed while underscoring the value of tailored reference data in small ruminant veterinary medicine.

## DATA AVAILABILITY

The supplementary data can be available from the corresponding author upon reasonable request.

## AUTHORS’ CONTRIBUTIONS

NB: Sample collection, data recording, analysis, and interpretation. NB, AW, HW, and AF: Formal analysis, data curation, writing – review and editing. AF: Conceptualization. All authors have read and approved the final version of the manuscript.
